# Direct Determination of Phosphatase Activity from Physiological Substrates in Cells

**DOI:** 10.1371/journal.pone.0120087

**Published:** 2015-03-18

**Authors:** Zhongyuan Ren, Le Duy Do, Géraldine Bechkoff, Saida Mebarek, Nermin Keloglu, Saandia Ahamada, Saurabh Meena, David Magne, Slawomir Pikula, Yuqing Wu, René Buchet

**Affiliations:** 1 Université de Lyon, Villeurbanne, France; 2 Université Lyon 1, Villeurbanne, France; 3 INSA-Lyon, Villeurbanne, France; 4 CPE Lyon, Villeurbanne, France; 5 Institut de Chimie et Biochimie Moléculaires et Supramoléculaires, Villeurbanne, France; 6 CNRS UMR 5246, Villeurbanne, France; 7 State Key Laboratory of Supramolecular Structure and Materials, Jilin University Changchun, 130012, China; 8 Department of Biochemistry, Nencki Institute of Experimental Biology and Polish Academy of Sciences, 02–093 Warsaw, Poland; Brigham and Women's Hospital, Harvard Medical School, UNITED STATES

## Abstract

A direct and continuous approach to determine simultaneously protein and phosphate concentrations in cells and kinetics of phosphate release from physiological substrates by cells without any labeling has been developed. Among the enzymes having a phosphatase activity, tissue non-specific alkaline phosphatase (TNAP) performs indispensable, multiple functions in humans. It is expressed in numerous tissues with high levels detected in bones, liver and neurons. It is absolutely required for bone mineralization and also necessary for neurotransmitter synthesis. We provided the proof of concept that infrared spectroscopy is a reliable assay to determine a phosphatase activity in the osteoblasts. For the first time, an overall specific phosphatase activity in cells was determined in a single step by measuring simultaneously protein and substrate concentrations. We found specific activities in osteoblast like cells amounting to 116 ± 13 nmol min^-1^ mg^-1^ for PPi, to 56 ± 11 nmol min^-1^ mg^-1^ for AMP, to 79 ± 23 nmol min^-1^ mg^-1^ for beta-glycerophosphate and to 73 ± 15 nmol min^-1^ mg^-1^ for 1-alpha-D glucose phosphate. The assay was also effective to monitor phosphatase activity in primary osteoblasts and in matrix vesicles. The use of levamisole – a TNAP inhibitor- served to demonstrate that a part of the phosphatase activity originated from this enzyme. An IC_50_ value of 1.16 ± 0.03 mM was obtained for the inhibition of phosphatase activity of levamisole in osteoblast like cells. The infrared assay could be extended to determine any type of phosphatase activity in other cells. It may serve as a metabolomic tool to monitor an overall phosphatase activity including acid phosphatases or other related enzymes.

## Introduction

Among the enzymes having a phosphatase activity and releasing P_i_, tissue non-specific alkaline phosphatase (TNAP) performs indispensable, multisystemic functions in humans [1]. It is expressed with high levels in bones, liver and neurons. It is absolutely required for bone mineralization and also necessary for neurotransmitter synthesis. So far there is no direct methodological approach to determine phosphate in the living cells without the need of labelling. Most standard methods for measuring inorganic phosphate or pyrophosphate are based on coupled enzyme assays, colorimetric methods, conductance, or radioactivity labeling which do not allow one-step determination in the cells. Protein determination and activity measurements must be performed separately. For example, the screening of TNAP inhibitors relied on the determination of the recombinant TNAP activity at alkaline pH using para-nitrophenylphosphate (*p*NPP), which is not a physiological substrate. A 1,000-fold more sensitive and 10-fold faster than the *p*NPP assay has been developed with *p*NPP dioxetane-based substrate [2–3]. However, TNAP inhibitors selected by using non physiological substrates may act differently on the living cells. Therefore, there is a strong demand for flexible and fast strategies to select inhibitors with improved prospects for clinical success [4]. Infrared (IR) spectroscopy [5] has been employed to identify specific finger-like signatures in microbial cells [6–10] in tumour cells [11–16] or in tissues [17–19] giving information of overall structural and biochemical cell composition or changes induced by anti-tumour drugs in cells [20–28]. Alkaline phosphatase activity in sera [29] has been determined by IR, suggesting the possibility of using IR for quantitative determination of an enzymatic activity in whole cells. Here, we report a continuous IR assay to determine phosphatase activity in osteoblasts and in matrix vesicles released by chondrocytes, characterized by high TNAP activity. We provided the proof of concept that infrared spectroscopy is a reliable assay to determine a phosphatase activity in the osteoblasts. For the first time, an overall specific phosphatase activity in a single step by measuring simultaneously protein and substrate concentrations was determined.

## Materials and Methods

### Isolation of matrix vesicles

Matrix vesicles (MVs) were prepared according to Wuthier’s method [30]. Femurs from twenty 17-day-chicken embryos were taken. Slices (1–3 mm thick) of growth plates and ephyseal cartilage were cut. They were washed five times in synthetic cartilage lymph (SCL) buffer containing 1.42 mM NaH_2_PO_4·_H_2_O, 1.83 mM NaHCO_3_, 12.7 mM KCl, 0.57 mM MgCl_2_, 5.55 mM D-glucose, 63.5 mM sucrose, 16.5 mM TES, 100 mM NaCl, 0.57 mM Na_2_SO_4_, pH 7.4. The slices were incubated at 37°C for 180 min by mixing continuously in SCL buffer containing 200–500 units g^-1^ tissue of type I collagenase from *Clostridium histolyticum* (Sigma) and 1 mM CaCl_2·_H_2_O. Then, it was filtered through a nylon filter. The filtrate was centrifuged at 600 g for 15 min at 4°C. After the first centrifugation the debris were discarded and the supernatant was centrifuged at 20 000 g for 20 min at 4°C. A second centrifugation at 80 000g for 60 min at 4°C was then performed. The supernatant was discarded and the pellet was gently washed with 1 mL Tris HCL (100 mM, pH 8.0) containing 5 μM ZnCl_2_ and 5 mM MgCl_2_. Washing medium was discarded and the intact pellet (around 0.05 mL) was suspended in 0–2 mL Tris HCL (100 mM, pH 8.0) containing 5 μM ZnCl_2_ and 5 mM MgCl_2_ (called throughout buffer A). The protein concentration in MVs was determined by Bradford [31] was in the range of 0.2 to 8 mg MV protein mL^-1^. MVs were freshly prepared for the IR measurements.

### Saos-2 Cells

Human osteosarcoma Saos-2 cells (ATCC HTB-85) were cultured in DMEM medium supplemented with 100 U mL^-1^ penicillin, 100 g mL^-1^ streptomycin (both from Sigma) and 10% FBS (v:v, Gibco). Stimulation of Saos-2 cells was induced by culturing the confluent cells in growth medium supplemented with 50 μg mL^-1^ ascorbic acid (AA) (Sigma) and 7.5 mM β-glycerophosphate (Sigma). After a six-day incubation, Saos-2 cells were detached with trypsin (Sigma) and 1 mM CaCl_2·_H_2_O. The cells were washed with 1 mL buffer A. After centrifugation, the supernatant was discarded. The washing and centrifugation procedures were repeated three times so that the DMEM buffer and trypsin were completely removed. An aliquot of 0.5 to 1.5 mg of freshly prepared Saos-2 cells as determined by weighting was taken and kept in Eppendorf tubes for the IR measurements. The cells were freshly prepared for IR measurements.

### Primary cell cultures from mice

All experiments were carried out according to the guidelines laid down by the French Ministère de l’Agriculture (n° 87–848) and the E.U. Council Directive for the Care and Use of Laboratory Animals of November 24th, 1986 (86/609/EEC). Animal experiments were performed under the authorization n°69–266–0501 (INSA-Lyon, DDPP-SV, Direction Départementale de la Protection des Populations—Services Vétérinaires du Rhône), according to the guidelines laid down by the French Ministère de l’Agriculture (n° 87–848) and the E.U. Council Directive for the Care and Use of Laboratory Animals of November 24th, 1986 (86/609/EEC). MLC (n°692661241), AG (n°69266332) and COS (n°69266257) hold special licenses to experiment on living vertebrates issued by the French Ministry of Agriculture and Veterinary Service Department. The experiments were realized on euthanized animals by dislocation of cervical vertebra, which didn’t require surgery and were not painful. This study was specifically approved by the Committee on the Ethics of Animal Experiments of the INSA of Lyon CETIL (permit Number: 012012). The preparation of primary cells were performed at INSA of Lyon, IMBL Building, 69100 Villeurbanne, France. Primary osteoblast cells were enzymatically isolated from calvaria (frontal and parietal bones) of 5–6 days old mice (C57BL/6J strain). Calvaria were dissected aseptically and cells were isolated using sequential digestion at 37°C with trypsin/EDTA 0.05% during 20 min and then with liberase 0.8 U mL^-1^ during 20 min. The first two digests were discarded, and cells obtained after two 45 min digestions with liberase 0.8 U mL^-1^ were collected, pooled and then filtered through a 100 μm cell strainer. The cells were plated at a density of 1 × 10^5^ cells per well in 22.1 mm culture dish in DMEM containing 15% FBS (v:v, Gibco), 100 U mL^-1^ penicillin and 100 μg mL^-1^ streptomycin (both from Sigma) and switched 24h later to growth medium i.e., DMEM containing 10% FBS (v:v, Gibco), supplemented with 50 μg mL^-1^ ascorbic acid (Sigma). After six days of culture, the medium was further supplemented with 7.5 mM β-glycerophosphate (Sigma) and 50 μg mL^-1^ ascorbic acid during one week. Primary cultures were used without passage. Cultures were maintained in a humidified atmosphere consisting of 95% air/5% CO_2_ at 37°C. After a six-day incubation, primary cells were detached with trypsin (Sigma) and 1 mM CaCl_2·_H_2_O. The cells were washed with 1 mL buffer A or with 1 mL DMEM buffer. After centrifugation, the supernatant was discarded. The washing and centrifugation procedures were repeated three times so that trypsin was completely removed. An aliquot of 0.5 to 1.5 mg of freshly prepared osteoblasts as determined by weighting was taken and kept in Eppendorf tubes for the IR measurements. The cells were freshly prepared for IR measurements.

### Infrared spectra

To start the reaction, an aliquot of either MV (0.5 to 8 mg protein mL^-1^), Saos-2 cells (6 to 28 mg protein mL^-1^) or primary osteoblast (20 to 45 mg protein mL^-1^) was taken and mixed with the reaction medium containing 40–80 mM final PP_i_ or one of other substrates (AMP, ADP, ATP, UTP, α-D-glucose 1-phosphate (G-1P), β-glycerophosphate (β-GP) and para-nitrophenylphosphate (*p*NPP)) in buffer A. Final protein concentrations in MVs or in cells were determined directly by measuring the intensity of the amide-II band at 1550-cm^-1^ with the concentration absorption coefficient ε = 3.6 mg^-1^mL cm^-1^. ε was determined by using bovine serum albumin as a protein Five μL of the reaction mixture containing either MVs or Saos-2 cells or primary osteoblast cells were taken and deposited between two BaF_2_ windows of a demountable thermostated cell (model Harrick) separated with a 12 μm (for IR spectra of substrates) or 6 μm (for IR spectra of cells with substrates) Teflon spacer. IR data were acquired with a Thermo Scientific Nicolet iS10 spectrometer equipped with a DTGS detector. The IR spectra were recorded with a thermostated IR cell kept at 37°C with 128 interferograms at 4 cm^–1^ resolution each and then Fourier transformed. During data acquisition, the spectrometer was continuously purged with dry filtered air (Balston regenerating desiccant dryer, model 75–45 12 VDC). At least three independent measurements have been performed to obtain the kinetics parameters. To minimize cell to cell variation, affecting TNAP activity, the same cell preparations were used to compare with the controls. For the determination of IC_50_ of levamisole, relative activities were measured to take into account of the cell to cell variations.

## Results and Discussion

### IR spectra of phosphatase substrates

**Fig 1:**
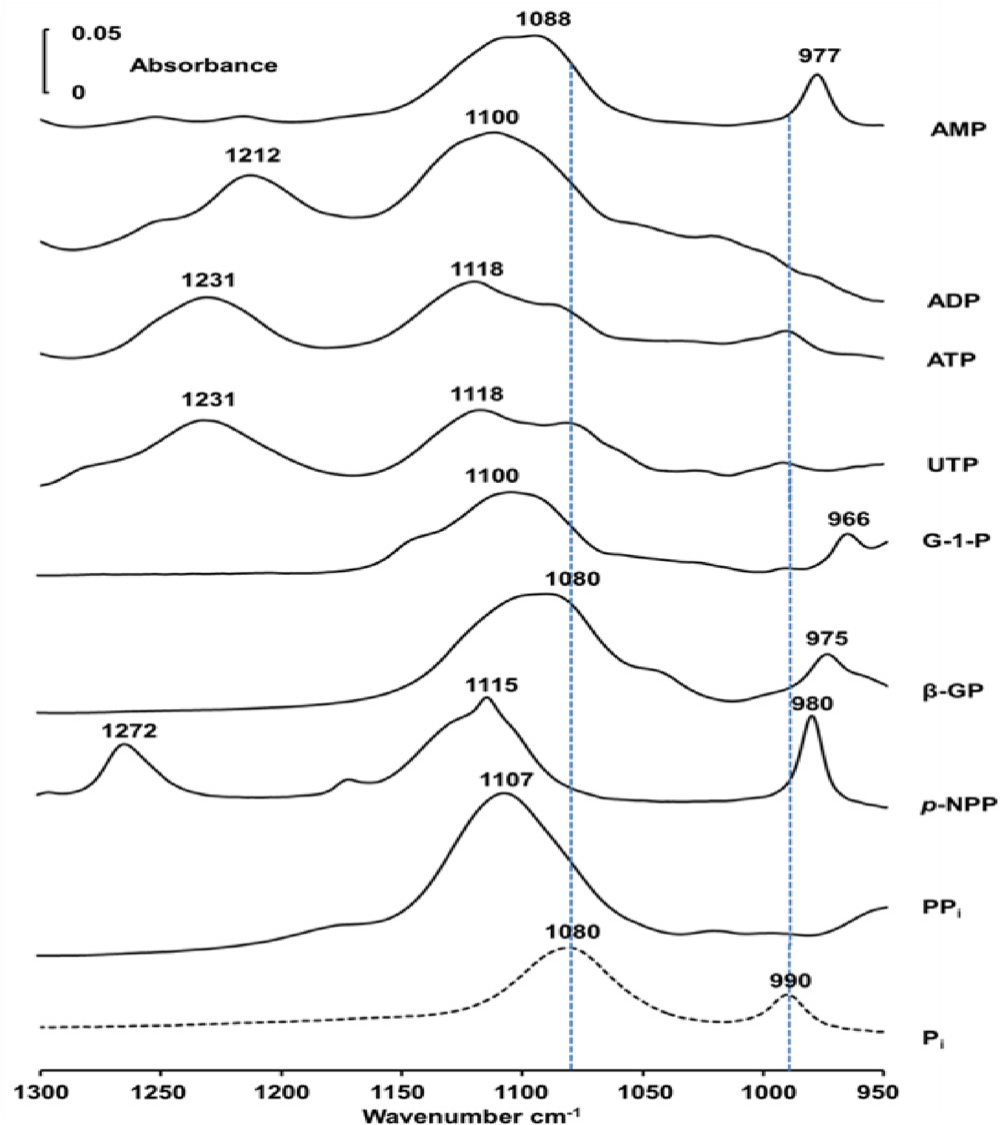
Infrared spectra of substrates. IR spectra of 50 mM phosphatase substrates: AMP, ADP, ATP, UTP, glucose-1-phosphate (G-1P), β-glycerophosphate (β-GP), *p*-nitrophenylphosphate (*p*-NPP), PP_i_ and P_i_ in buffer A (100 mM Tris-HCl pH 8.0, 5 μM ZnCl_2_ and 5 mM MgCl_2_) recorded at 37°C.

IR spectra of phosphatase substrates such as AMP, ADP, ATP, UTP, α-D-glucose 1-phosphate (G-1P), β-glycerophosphate (β-GP), *p*NPP and PP_i_ in aqueous Tris-HCl buffer (pH 8) containing 5 mM MgCl_2_ and 5 μM ZnCl_2_ (buffer A) present sufficient differences in band shapes and positions as compared with the IR spectrum of phosphate (Fig. 1) to be used for analytical application.

**Fig 2:**
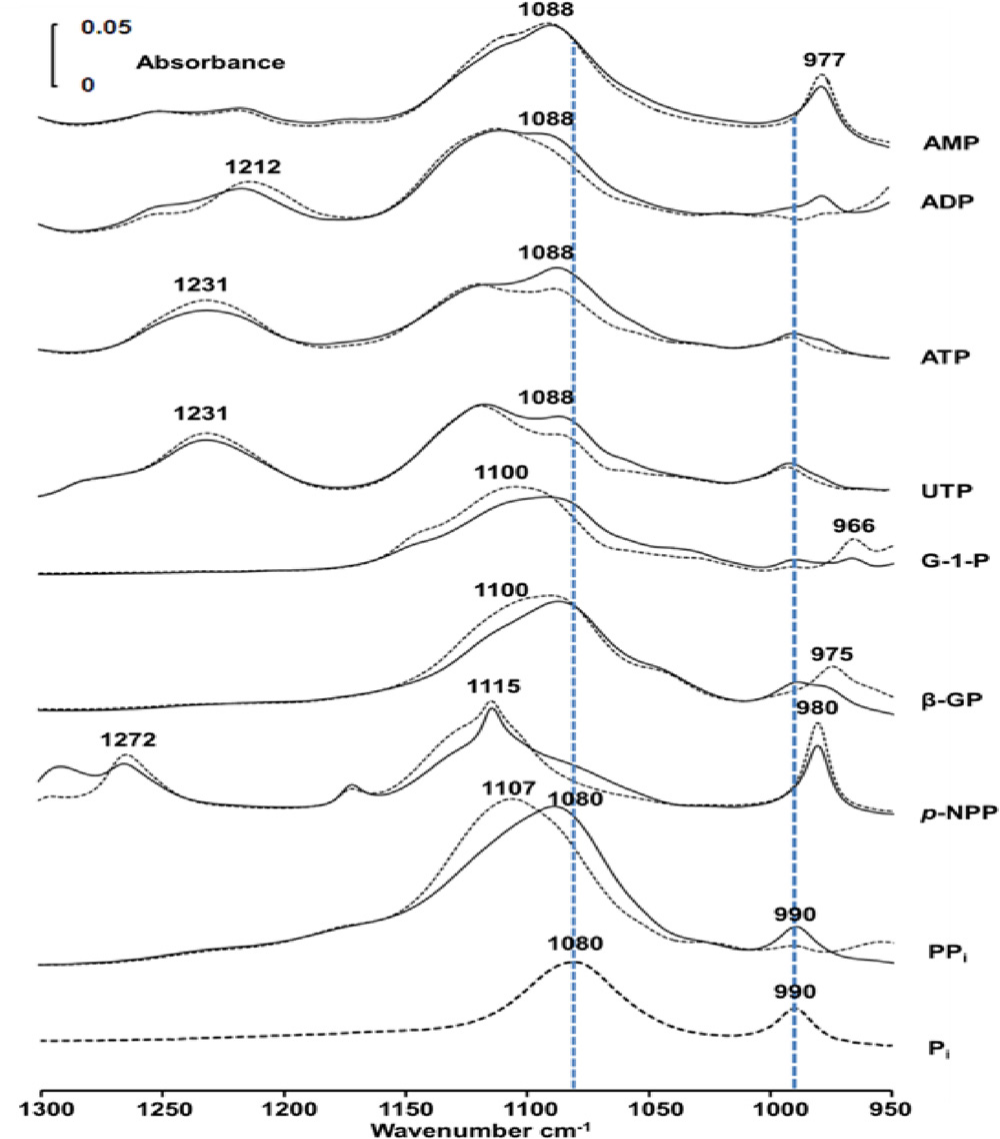
Infrared spectra of hydrolyzed substrates in osteoblast-like cells. IR spectra of 50 mM substrate (one among the substrates: AMP, ADP, ATP, UTP, G-1P, β-GP, *p*NPP, PP_i_ and P_i_) in buffer A containing Saos-2 cells (6–15 mg of Saos-2-cell protein mL^-1^), recorded immediately after mixing the substrate with cells (dashed lines) and after 30 min (full lines) at 37°C. The bottom trace shows the spectrum of 50 mM P_i_ to indicate the positions of the two 1076–1080 and 990 cm^-1^ bands.

The IR spectrum of P_i_ in buffer A revealed two bands at 1076 and 990 cm^-1^ (Fig. 1) that are assigned respectively to asymmetric and symmetric stretching vibrations of O-P-O, respectively [32]. The position of the phosphate bands is sensitive to their ionic environment as in the case of nucleotides [33] or phospholipids [34,35]. To illustrate the potential of the IR assay in assessing phosphatase activity in cells, we measured IR spectrum immediately (with 3 min delay) after mixing 50 mM substrate with osteoblast-like Saos-2 cells, (Fig. 2, dashed lines) and after 30 min of incubation (Fig. 2, full lines).

**Fig 3:**
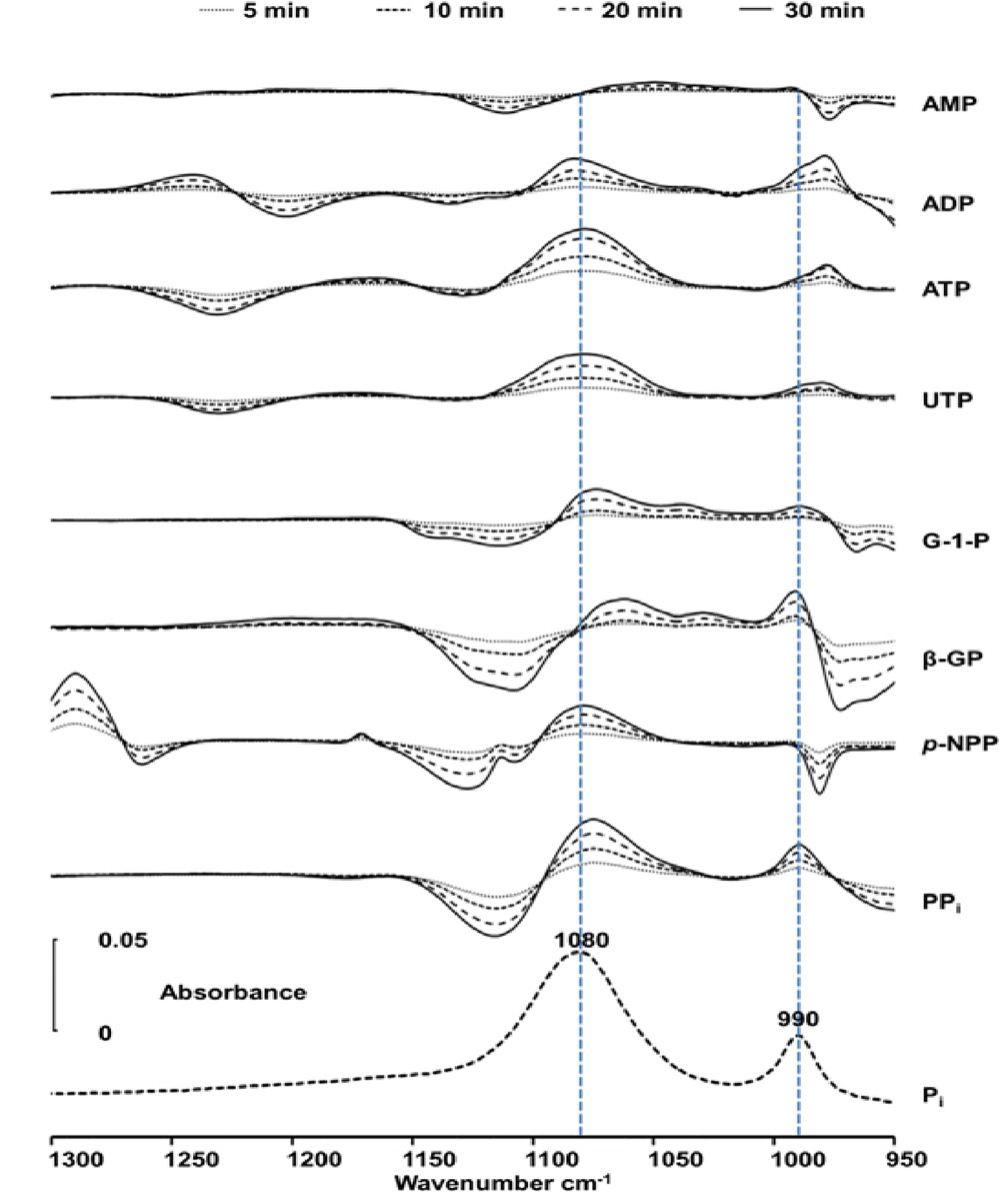
Infrared difference spectra of hydrolyzed substrates in osteoblast-like cells. Successive difference spectra of Saos-2 cells with 50 mM substrate (AMP, ADP, ATP, UTP, G-1P, β-GP, pNPP and PP_i_) in buffer A (spectrum measured after a given time minus that measured immediately after mixing the substrate with cells) at 37°C determined at 5, 10, 20 and 30 min. The negative intensities indicated the decline of substrate concentration while the positive intensities of the 1076–1080 and the 990 cm^-1^ bands indicated the appearance of P_i_. The bottom trace shows the spectrum of 50 mM P_i_ to show the positions of the two 1076–1080 and 990 cm^-1^ bands.

In all cases (with AMP, ADP, ATP, UTP, G-1P, β-GP), *p*NPP or PP_i_), the intensity of one or both P_i_ bands at 1076–1080 and 990 cm^-1^ increased, indicating the phosphatase activity in the whole cells (Fig. 2). It was clearly observed in the successive difference spectra (spectrum measured at the indicated time minus that measured immediately after mixing 50 mM substrate with Saos-2 cells) (Fig. 3).

**Fig 4:**
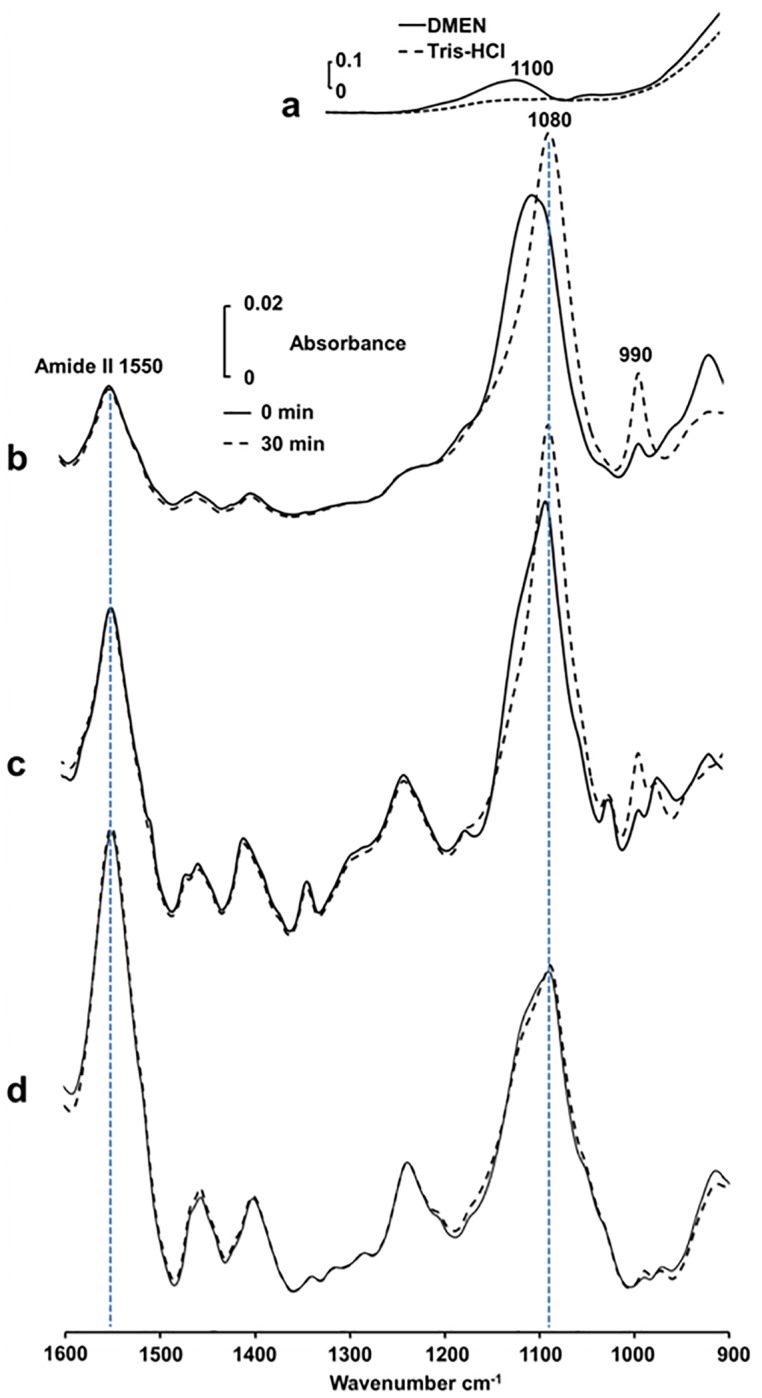
Infrared spectra of PP_i_ during its hydrolysis in osteoblast-like cells and in primary cells. (a) IR spectrum of buffer A and IR spectrum of DMEM. (b) IR spectra of Saos-2 cells (12 mg of Saos-2-cell protein mL^-1^) with 50 mM PP_i_ in buffer A recorded immediately after mixing the substrate with cells (dashed line) and after 30 min incubation at 37°C (full line). The spectrum of buffer A was deducted from the spectra of Saos-2 cells. (c) IR spectra of Saos-2 cells (28 mg of Saos-2-cell protein mL^-1^ with 50 mM PP_i_ in DMEM recorded immediately after mixing the substrate with cells (dashed line) and after 30 min incubation at 37°C (full line). The spectrum of DMEM was deducted from the spectra of Saos-2-cells. (d) IR spectra of primary osteoblast cells (45 mg of cell protein mL^-1^) in buffer A with 50 mM PP_i_ recorded immediately after mixing PP_i_ with cells (dashed line) and after 30 min incubation at 37°C (full line). The spectrum of buffer A was deducted from the spectra of osteoblasts.

Negative peaks indicated the disappearance of the substrate while positive peaks at 1076–1080 and 990 cm^-1^ illustrated the formation of P_i_ (Fig. 3). Hydrolysis of ATP, UTP and ADP in the Saos-2 cells produced P_i_ (positive 1088–1080-cm^-1^ and 990-cm^-1^ bands) and AMP (positive 977-cm^-1^ band) (Fig. 3). In the case of AMP hydrolysis, the P_i_ bands (located 1088–1080 cm^-1^ and 990 cm^-1^) overlapped with the AMP bands (located at 1088 cm-1 and 977 cm-1) rendering difficult to observe the Pi bands. We also observed hydrolysis of G-1P, β-GP and *p*NPP and PP_i_ in Saos-2 cells indicating a broad phosphatase activity. Buffer A has the disadvantage of inducing cell aggregation after several hour incubation but its absorption in the 1200–1000-cm^-1^ region (Fig. 4a, dashed line) is lower than that of DMEM cellular medium (Fig. 4a, full line).

**Fig 5:**
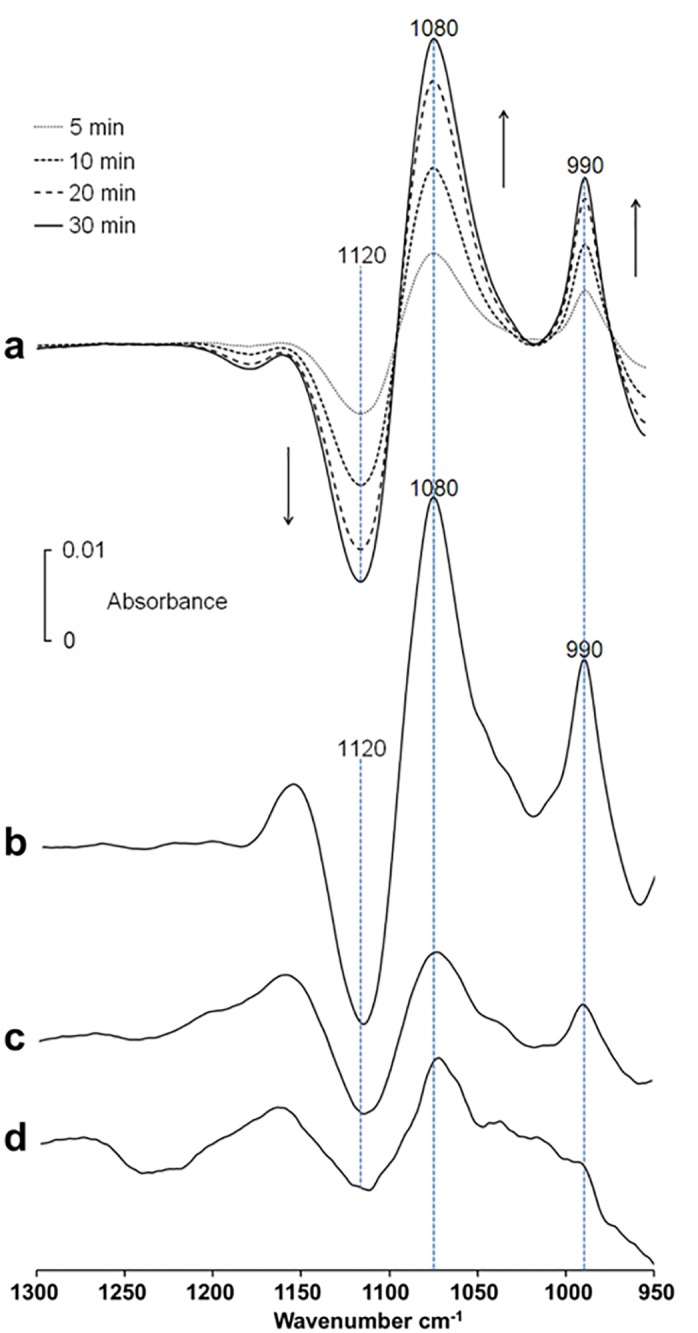
Infrared difference spectra of PP_i_ during its hydrolysis in osteoblast-like cells and in primary cells. (a) Successive IR difference spectra of Saos-2 cells (12 mg of Saos-2-cell protein mL^-1^) in buffer A with 50 mM PP_i_ at 37°C from 5 min to 30 min (spectrum recorded after the indicated time minus spectrum recorded immediately after mixing substrate with cells). (b) IR difference spectrum of Saos-2 cells (28 mg of cell protein mL^-1^) with 50 mM PP_i_ in DMEM medium at 37°C (spectrum recorded after 30 min minus spectrum recorded immediately after mixing substrate with cells). (c) IR difference spectrum of primary osteoblast cells (45 mg of cell protein mL^-1^) with 50 mM PP_i_ in buffer A at 37°C (spectrum recorded after 30 min minus spectrum recorded immediately after mixing substrate with cells). (d) IR difference spectrum of primary osteoblast cells (220 mg of cell protein mL^-1^) with 50 mM PP_i_ in DMEM at 37°C. (spectrum recorded after 30 min minus spectrum recorded immediately after mixing substrate with cells).

The feasibility to monitor the hydrolysis of PP_i_ by Saos-2 cells in DMEM was verified. Typical IR spectra of 50 mM PP_i_ with Saos-2 cells in buffer A (Fig. 4b) or in DMEM (Fig. 4c) immediately after mixing PP_i_ with Saos-2 cells (full trace) and after 30 min incubation (dashed trace) indicated both the decrease of the 1100-cm^-1^ band of PP_i_ and the increase of the 1076–1080-cm^-1^ band of P_i_, confirming the PP_i_ hydrolysis. Furthermore, the 1550-cm^-1^ protein band allowed us to determine directly protein concentration using the concentration absorption coefficient ε = 3.6 mg^-1^mL cm^-1^. Saos-2 osteoblast-like cells were selected due to their high TNAP activity, making it easier to monitor phosphatase activity. However they have different phenotypic properties in comparison with the corresponding healthy osteoblasts. Primary osteoblasts from mouse calvaria exhibited also PP_i_ hydrolysis both in buffer A (Fig. 4d) and in DMEM but it was much lower than that of Saos-2 cells. This was confirmed qualitatively by the magnitude of the difference in IR spectra (spectrum measured after 30 min minus that measured immediately after mixing PP_i_ with cells) recorded for Saos-2 cells in buffer A (Fig. 5a) and in DMEM medium (Fig. 5b) which were higher than that those of primary osteoblasts in buffer A (Fig. 5c) and in DMEM (Fig. 5d) respectively.

### Determination of specific activity of phosphatase in Saos-2 cells

**Fig 6:**
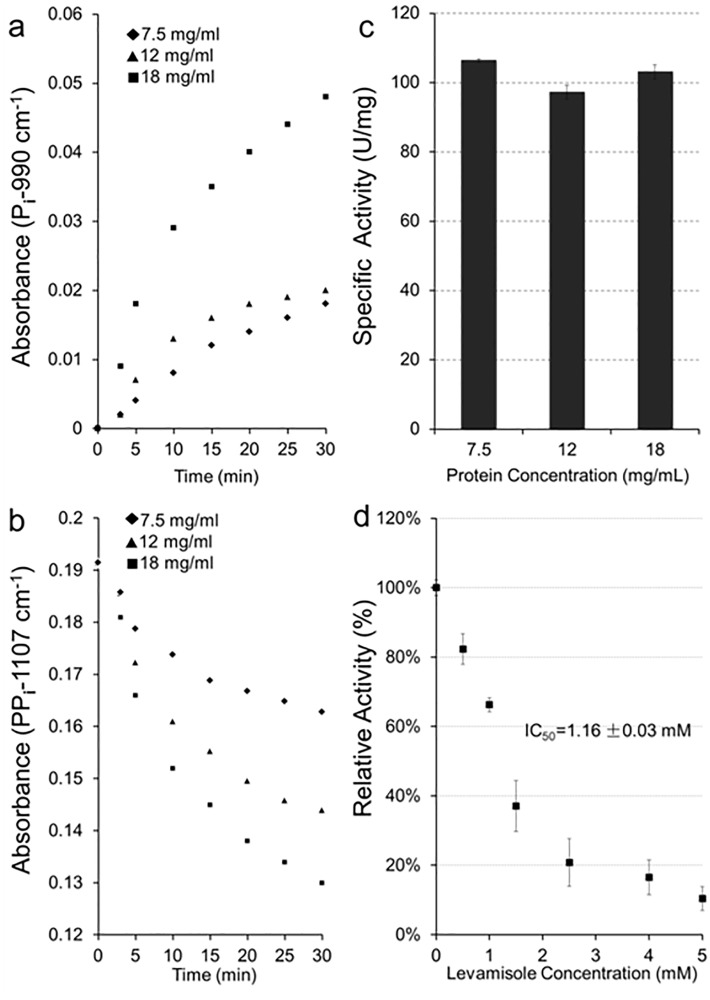
Determinations of phosphatase activity in Saos-2 cells and IC_50_ of levamisole. (a) Absorption of PP_i_-band as a function of incubation time and of Saos-2 cell protein concentration (from 7.5 to 18 mg mL^-1^). (b) Absorption of PP_i_ in 1120–1100-cm^-1^ region as a function of incubation time and of Saos-2 cell protein concentration. (c) Specific activity of PP_i_ hydrolysis by Saos-2 cells at three concentrations from 7.5, 12 and 18 mg mL^-1^. One enzyme unit (1 U) is expressed as 1 nmol hydrolyzed PP_i_ per min at 37°C. As expected, the specific activity values determined as function of protein concentration in cells were not significantly different from each other. (d) Determination of IC_50_ of levamisole by measuring inhibition of PPi hydrolysis by Saos-2 cells.

The kinetic parameters are easily obtained from IR spectra. For example, the PP_i_ hydrolysis by Saos-2 cells is followed by an increase of the 990-cm^-1^ band of P_i_ (Fig. 6a) and the concomitant decrease of the 1120–1100-cm^-1^ band of PP_i_ (Fig. 6b).

The absorbance changes of 1120–1100-cm^-1^ band of PP_i_ (Fig. 6b) and the 990-cm^-1^ band of P_i_ (Fig. 6a) served to determine the PP_i_ hydrolysis (Table 1).

**Table 1:** Specific activity of phosphatase activity of several substrates by osteoblast-like Saos-2 cells in buffer A at 37°C as determined by the infrared band of substrate or Pi (990 and 1070 cm^-1^).

Substrates	Band position cm^-1^ (molecule)	Mol. Abs. coeff. L.mol^-1^.cm^-1^	Specific activity nmol.mg^-1^min^-1^	Average nmol.mg^-1^.min^-1^
AMP	977 (AMP)	476 ± 48	66 ± 4	
				58 ± 4*
	990 (P_i_)	443 ± 50	42 ± 7	
G-1-P	990 (P_i_)	443 ± 50	119 ± 22	
				106 ± 15
	1080 (P_i_)	1215 ± 131	92 ± 12	
β-GP	990 (P_i_)	443 ± 50	97 ± 2	97 ± 2
*p*-NPP	980 (*p*-NPP)	1210 ± 81	108 ± 3	
				121 ± 13
	1080 (P_i_)	1215 ± 131	135 ± 4	
PP_i_	1107 (PP_i_)	2158 ± 21	116 ± 22	
	990 (P_i_)	443 ± 50	127 ± 12	118 ± 10
	1080 (P_i_)	1215 ± 131	110 ± 4	

(*) Specific activity of TNAP using AMP was significantly different from that of TNAP using PP_i_ (p = 0.0004).

As expected, increasing the amount of Saos-2 cells, from 7.5 mg mL^-1^, 12 mg mL^-1^ to 18 mg mL^-1^, as determined by the protein band, increased the PP_i_ hydrolysis but the specific activity remained at the level of 102 ± 4 nmol min^-1^ mg^-1^ as expected (Fig. 6c and Table 1). There is not a statistically significant difference between the specific activities (P value is at least greater than 0.513). This compares well with the specific PP_i_ hydrolysis of 130 nmol min^-1^ mg^-1^ in mammalian HeLa cells at pH 8.5 (36) as assayed by the modified method of Fiske and Subbarow. Quantitative determinations were also obtained for AMP, G-1P, β-GP, *p*NPP using both P_i_ bands at 990 and 1076–1080 cm^-1^ (Table 1). The substrate bands were also used to determine enzyme specific activity, as in the case of AMP (977 cm^-1^), *p*NPP (980 cm^-1^) and PP_i_ (1107 cm^-1^) (Table 1). Specific activities determined either from P_i_ or from substrate-bands were identical within experimental errors except for G-1P and β-GP due to strong band overlapping. To check if the pyrophosphatase activity originates from TNAP-enriched cells, levamisole (a TNAP inhibitor) was added to the medium. At the concentration of 5 mM, levamisole almost completely inhibited the PP_i_ hydrolysis by Saos-2 cells (Fig. 6d), suggesting that TNAP contributed mostly to the PP_i_ hydrolysis of Saos-2 cells. Relative activity instead of specific activity was used to take into account of cell variability. 100% corresponded to the specific activity without the addition of inhibitor. An IC_50_ value of 1.16 ± 0.03 mM was obtained for the inhibition of phosphatase activity of levamisole which is comparable with the reported concentration of 1 mM levamisole that blocked calcification in cultures of aortas from uremic rats [37].

### Determination of specific activity of phosphatase in matrix vesicles

**Fig 7:**
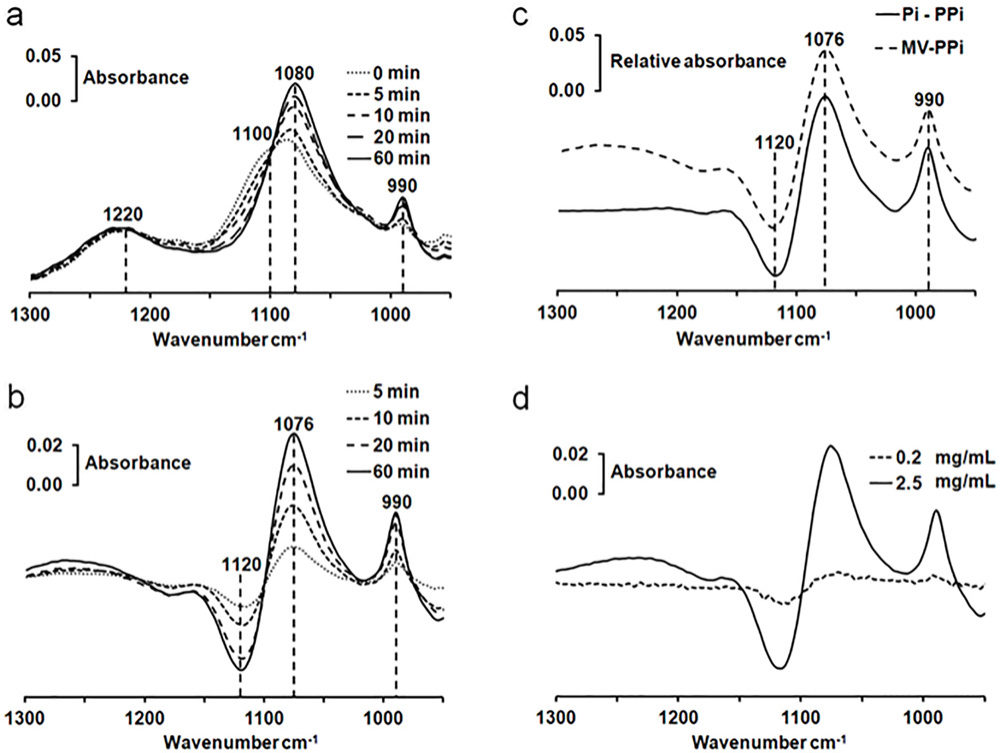
Phosphatase activity in matrix vesicles as determined by infrared spectroscopy. (a) Kinetic recording of 50 mM PP_i_ hydrolysis by 2 mg MV protein mL^-1^ in buffer A at 37°C. Successive infrared spectra were measured at the indicated times. (b) Difference IR spectra of MVs with PP_i_ (spectrum recorded at the indicated time minus that recorded immediately). Significant variation in the intensity of absorption bands of PP_i_ located at 1120–1100 cm^-1^ as well as other variations attributed to P_i_ at 1076–1080 cm^-1^ and 990 cm^-1^ were observed. (c) Expected IR difference spectrum of product and substrate (2xP_i_ minus PP_i_) (dashed line). IR difference spectrum of the hydrolysis of 50 mM PP_i_ by 2.5 mg MV protein mL^-1^ in buffer A (spectrum recorded at 60 min minus spectrum recorded immediately) (Full line) d) Sensitivity of the infrared assay to determine the hydrolysis of 50 mM PP_i_ by 2.5 mg MV protein mL^-1^ (full line) and by 0.2 mg MV protein mL^-1^ (dashed line) as shown by their difference spectrum respectively (spectrum of MVs with 50 mM PP_i_ recorded at 30 min minus spectrum recorded immediately).

Matrix vesicles (MVs) extracted from growth plates and epiphyseal cartilage of 17-day-chicken embryos were also assayed for PP_i_ hydrolysis since they have 10–40 fold higher TNAP specific activity than cells. MV size is around 100–400 nm diameter as determined by electron microscope [38]. Typical example of a series of IR spectra during PP_i_ hydrolysis of MVs in Buffer A supplemented with 50 mM PP_i_ monitored at 37°C are shown in Fig. 7a.

**Fig 8:**
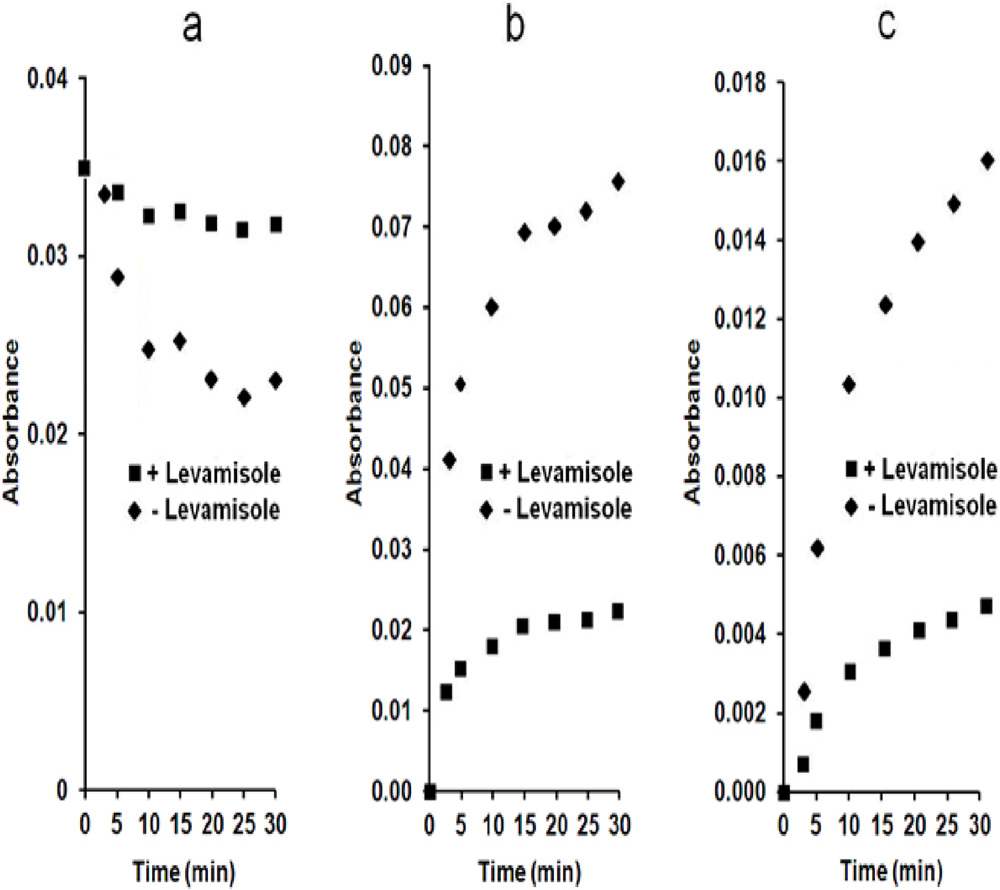
Inhibition of pyrophosphatase activity in matrix vesicles by levamisole. (a) Absorption changes of PP_i_ band at 1107 cm^-1^; (b) Absorption changes of P_i_ peak at 990 cm^-1^; (c) Absorption changes of P_i_ band at 1076 cm^-1^ during PP_i_ hydrolysis by 2 mg mL^-1^ MVs in buffer A at 37°C in the absence (◆) or presence (∎) of 5 mM levamisole.

The intensity decrease of the 1120–1100 cm^-1^ band related to the disappearance of PP_i_ is correlated with the increase in the 1076–1080 and 990 cm^-1^ bands (Fig. 7a), indicating a hydrolysis of PP_i_ by MVs and an appearance of P_i_. It is better seen in the difference infrared spectra, e.g. from the IR spectrum of MVs recorded at the indicated time minus that recorded immediately (Fig. 7b), which were similar with difference spectra of Saos-2 cells with PP_i_ (Fig. 3) and also with those of alkaline phosphatase with PP_i_ (data not shown). The calculated IR difference, i.e. spectrum of P_i_ minus spectrum of PP_i_ (Fig. 7c, dashed line) is almost identical to the IR difference spectrum of MVs with PP_i_ (spectrum recorded after 60 min minus the spectrum recorded immediately) (Fig. 7c, full line). The IR changes produced by PP_i_ hydrolysis by MVs corresponded to the calculated decrease of one PP_i_ and to the formation of two P_i_. We estimated the sensitivity of the IR assay during 30-min incubation to be about 0.2 mg mL^-1^ MV protein. At 0.2 mg mL^-1^ MV protein, only faint bands not much more intensive than the noise signal are detected (Fig. 7d, dashed line), while at 2.5 mg mL^-1^ MV protein, the IR bands indicating disappearance of PP_i_ and appearance of P_i_ are well resolved (Fig. 7d, full line). Activity measurements were determined by measuring absorbance changes of the absorbance changes of PP_i_ band located at 1107 cm^-1^ using ε = 2158 ± 211 M^-1^ cm^-1^ (Fig. 8a) as well as with two P_i_ bands located at 990 cm^-1^ (Fig. 8b) and 1076 cm^-1^ (Fig. 8c), using ε = 443 ± 50 M^-1^ cm^-1^ and ε = 1215 ± 131 M^-1^ cm^-1^, respectively. As a result we determined an apparent hydrolytic activity of MVs towards PP_i_ of 1132 ± 370 nmol min^-1^ mg^-1^.

For comparison, the TNAP activity of MVs determined at pH 10.4 using the *p*NPP as a substrate, amounted to 30 000 ± 10 000 nmol min^-1^ mg^-1^. This is consistent with the reported values for alkaline phosphatase extracted from HeLa cells, having a PP_i_ hydrolytic activity at pH 8.5 about 26 to 30 times smaller than the *p*NPP hydrolytic activity at pH 10.5 [36]. To test if the hydrolysis of PP_i_ is specifically associated to TNAP, we incubated the reactive medium containing MVs and 50 mM PP_i_ with 5 mM levamisole, a TNAP specific inhibitor. Addition of levamisole induced a smaller slope in the absorbance changes at 1107 cm^-1^ (Fig. 8a), at 990 cm^-1^ (Fig. 8b) and at 1076 cm^-1^ (Fig. 8c) as compared with the control sample without levamisole (Fig. 8). We observed a significant inhibition of PP_i_ hydrolysis in the presence of 5 mM levamisole from 1132 ± 370 nmol min^-1^ mg^-1^ (without levamisole) to 343 ± 112 nmol min^-1^ mg^-1^ (with 5 mM Levamisole) confirming that TNAP is to a large extent responsible for PP_i_ hydrolysis. Since the levamisole could not block completely the phosphatase activity, there is the possibility that other phosphatases such as NPP1 can catalyze the phosphatase activity. Indeed, NPP1, which usually hydrolyses phosphodiester bond can also hydrolyze phosphomonoester bond at a lesser extend [39].

## Conclusion

It has been recognized earlier that IR spectroscopy has considerable potential as a tool in diagnostics since it may be used to assess biochemical changes of cells exposed to various inhibitors [20–28]. Here we demonstrated the ability of IR spectroscopy to directly determine *in situ* a phosphatase activity in osteoblasts using natural substrates without any labeling. We showed the ability of IR spectroscopy to directly determine simultaneously protein concentration and the phosphatase activity in cultured osteoblasts as well as in matrix vesicles using physiological substrates such as AMP, ADP, ATP, UTP, and PP_i_. The method has been validated for osteoblasts (Saos-2 cells and primary osteoblasts) and chondrocyte-derived matrix vesicles which are all TNAP enriched. This approach could be extended to determine alkaline phosphatase activity as well as any type of phosphatase activity in other cells. It may serve as a metabolomic tool to determine an overall phosphatase activity in a cell which may involve several enzymes.
